# Half-turned truncal switch for transposition of the great arteries with left ventricular outflow tract obstruction

**DOI:** 10.1016/j.xjtc.2025.10.027

**Published:** 2025-11-11

**Authors:** Frank G. Scholl, Mark Ruzmetov, John N. Dentel, Steve Bibevski

**Affiliations:** Division of Pediatric Cardiothoracic Surgery, The Heart Institute, Joe DiMaggio Children's Hospital, Hollywood, Fla

**Keywords:** congenital heart disease, transposition of the great arteries, half-turned truncal switch, outcomes, Rastelli operation

## Abstract

**Objective:**

The Rastelli operation is accepted as 1 of the standard techniques for complete transposition of the great arteries (TGA) and TGA-type double-outlet right ventricle with left ventricular outflow tract (LVOT) obstruction. The half-turned truncal switch (HTTS) operation has been reported as an alternative to the Rastelli approach. The aim of this study was to compare these 2 operations for children with TGA and LVOT obstruction (TGA/LVOTO).

**Methods:**

Between 2012 and 2024, 11 patients underwent TGA/LVOTO repair. The median age at repair was 12 months and median weight was 8.4 kg. Preoperative baseline and follow-up data were collected from records. Diagnosis was TGA with pulmonary stenosis in 7 cases, TGA-type double-outlet right ventricle in 4 cases. One patient had a hypoplastic right ventricle. Rastelli operation was performed in 6 patients, and 5 patients underwent the HTTS operation. The right ventricular outflow tract was augmented using a CorMatrix monocusp valve patch in 4 patients during HTTS operation, and Contegra (Medtronic) (n = 5) or pulmonary homograft (n = 1) valve during Rastelli operation.

**Results:**

There were no early deaths; all patients survived the procedure. The groups did not differ significantly in gender, age/weight/height in complete repair, previous surgical or transcatheter procedures, postoperative length of stay, genetic/syndromic abnormalities, and crossclamp time during complete repair. The mean cardiopulmonary bypass time was significantly longer for patients after HTTS (HTTS, 264 ± 24 minutes vs Rastelli, 174 ± 57 minutes; *P* = .01). The mean follow-up time was 5.2 ± 4.8 years (range, 1 month to 12 years). There was no significant difference between groups in follow-up time, pulmonary stenosis gradient, or degree of pulmonary or aortic insufficiency. Conduit reintervention was required in 4 patients from the Rastelli group and none in the HTTS group (Rastelli, 67% vs HTTS, 0%; *P* = .06).

**Conclusions:**

Our study suggests that in children with TGA or TGA-type double-outlet right ventricle with LVOT obstruction, the half-turned truncal switch operation provides an unobstructed LVOT, results in less need for reintervention, and shows no disadvantage associated with right ventricular outflow tract growth at any anatomic level. Further investigation on this topic is needed.

**Figure:**
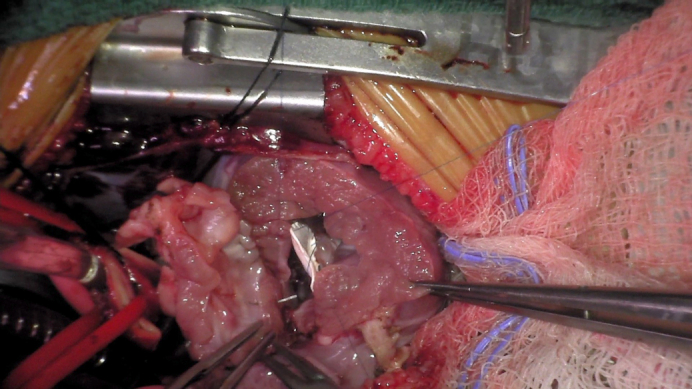
Suturing of the half-turned truncus to the left ventricular outflow tract.

**Central Message**

Half-turned truncal switch is the preferred treatment option at our institution for transposition of the great arteries with ventricular septal defect and left ventricular outflow tract obstruction.

**Perspective**

When compared with the Rastelli operation, the half-turned truncal switch technique may provide a preferred treatment option for transposition of the great arteries and double-outlet right ventricle with ventricular septal defect and left ventricular outflow tract obstruction due to improved left and right ventricular outflow tracts, avoidance of the conduit, and growth potential.

Surgical decision making and management of patients with D-transposition of the great arteries (TGA) and left ventricular outflow tract (LVOT) obstruction with ventricular septal defect (VSD) who are not amenable to standard arterial switch operation may present a significant challenge.

Generally, conventional surgical procedures for such cases are the Rastelli procedure,^1^ réparation à l'étage ventriculaire (REV) operation,^2^ and Nikaidoh procedure^3^; however, each of these options have drawbacks, including postoperative LVOT and right ventricular outflow tract (RVOT) obstruction or coronary insufficiency.^4^, ^5^, ^6^, ^7^ In the Rastelli procedure specifically, the systemic blood flow is directed from the systemic ventricle through the VSD into the aorta by an intracardiac baffle. This tunnel is often long and may lead to subaortic obstruction later in life depending on the anatomic substrate (length of the conal septum, size of the pulmonary root, size of the VSD, and distance of the VSD to the aortic root). Furthermore, an RV to pulmonary artery (RV-PA) conduit is necessary.^6^, ^7^, ^8^ Yamagishi and colleagues^9^^,^^10^ developed the half-turned truncal switch (HTTS) operation to overcome these drawbacks. In this operation, the harvested truncal block, including both semi-lunar valves, are anastomosed to the appropriate outflow tracts (the LV to the aortic valve, and the RV to the PA after horizontal half-turning).^11^ This allows for a relatively straight, unobstructed pathway from the LV to the aorta with minimal opportunity for future narrowing. Additionally, the pulmonary semi-lunar valve can be augmented with a monocusp and patch to provide unobstructed RVOT and maintain competence. The aim of this study was to evaluate and compare the use of these 2 types of operations at a single center for children with TGA/LVOT obstruction.

## Materials and Methods

### Study Patients

Following institutional review board exemption, the Joe DiMaggio Children's Hospital Heart Institute's Cardiology and Cardiac surgery databases were searched for children undergoing Rastelli or HTTS operative repair from January 2012 to December 2024. Written consent was obtained, including a statement stating all records could be used for scientific and educational purposes inclusive of publication.

We identified 11 patients who underwent TGA/LVOT obstruction repair. The median age at repair was 12 months (range, 49 days to 5.6 years) and median weight was 8.4 kg (range, 4-14.5 years). Preoperative baseline and follow-up data were collected from records. Diagnosis was TGA with pulmonary stenosis (PS) in 7 cases and TGA-type double-outlet right ventricle with PS in 4 cases. The Rastelli operation was performed in 6 patients, and 5 patients underwent the HTTS. The decision to perform a Rastelli operation or proceed with HTTS operation was determined based on surgeon experience/preference; coronary anatomy, with interarterial or complex coronary pattern crossing the outflow tract considered a contraindication to HTTS; and to a lesser degree on great vessel position. The degree of pulmonary hypoplasia or PS was not considered to be a factor and no patients had significant preoperative pulmonary regurgitation. Follow-up was 100% complete on all patients in both groups at our institution. Demographic data comparison is shown in Table 1.

**Table 1:** Demographic data comparison

Variable	Rastelli group (n = 6)	HTTS group (n = 5)	*P* value
Age (mo)	9.6 ± 5.4	16.5 ± 10	.23
Weight (kg)	7.1 ± 2.1	14.5 ± 9.8	.11
Height (cm)	64.2 ± 10.3	80 ± 10	.06
Gender			1.00
Male	4	3	
Female	2	2	
Chromosomal/syndromic abnormalities	0	2 (40)	.47
Prior intervention	4 (67)	2 (40)	.57

Values are presented as mean ± SD, n (%), or n. *HTTS*, Half-turned truncal switch.

A previous cardiac procedure was performed in 6 patients (55%): surgical Blalock-Taussig or central shunt in 4 patients, and transcatheter patent ductus arteriosus stenting in 2 patients. The relationship of the great arteries was anterior posterior in all but 1 patient (side-by-side). The coronary artery was Yacoub type A in 10 and type D in 1. In 1 patient, preoperative RV volume was <80% of the normal value. Pulmonary-aortic annular diameter ratio ranged from 0.47 to 1.00. In 1 case with an almost normal pulmonary annular diameter, a conventional arterial switch operation was deemed unlikely to be successful due to a dysplastic pulmonary valve. Using a complete pulmonary commissurotomy, the autologous pulmonary annulus was opened and preserved in 4 patients (HTTS group) with adequate annular diameter and flexible valve leaflets. The RVOT was then augmented using a CorMatrix monocusp valve patch in 5 patients during HTTS operation to fill in the space left after dividing the pulmonary (now anterior) leaflet, and Contegra (Medtronic) (n = 5) or pulmonary homograft (n = 1) valved conduit during Rastelli operation. Four patients (all from the HTTS group; n = 4 out of 11 [36%]) required delayed sternal closure and none of the patients in either group required extracorporeal membrane oxygenation support.

RVOT obstruction and LVOT obstruction were quantified using peak gradient calculation derived by Doppler interrogation. Degree of stenosis was qualified as mild (<30 mm Hg), moderate (30-50 mm Hg), or severe (>50 mm Hg). The presence and quantity of neopulmonary or neoaortic regurgitation were evaluated by color Doppler imaging and graded as none (0), trivial (I), mild (II), moderate (III), or severe (IV) depending on the ratio of the width of the regurgitant jet to the diameter of the LVOT.^12^

### Surgical Technique

**Figure 1:**
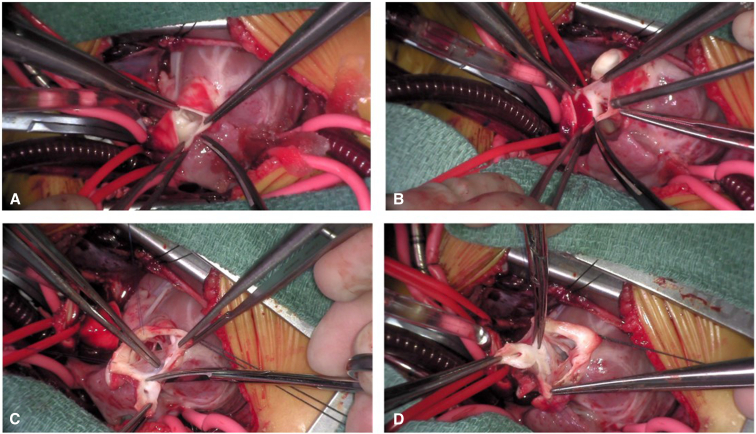
Dividing the aorta (A) and main pulmonary arteries (B). Harvesting the left (C) and right (D) coronary artery buttons. If necessary, the coronaries can be mobilized a bit farther away from the planned harvest incision to allow for more room for later reconstruction.

**Figure 2:**
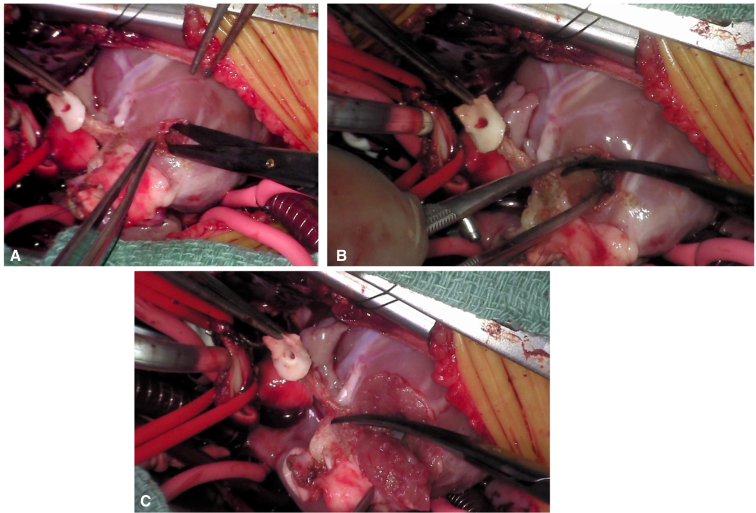
Harvesting the anterior and left side of the truncal block (A, B, and C). Thought should be given during the harvest to leave enough tissue on both sides of the incision to allow for the suturing of the half-turned truncal block during reconstruction. If necessary, the coronary artery can be mobilized a bit further away from the incision to allow for more room for reconstruction.

**Figure 3:**
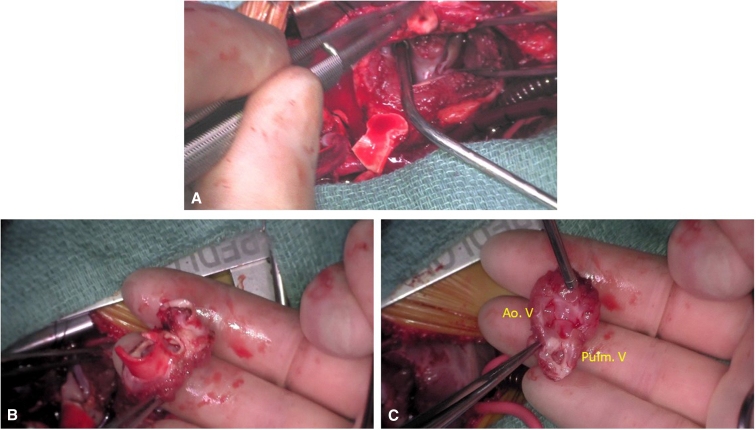
Type D coronary artery anatomy with circumflex coronary artery coursing posterior to the truncal harvest site (A). Although this anatomy is not a contraindication to the procedure, care should be taken to leave enough myocardium to suture the trunk back into place while thoughtfully avoiding injury to the circumflex coronary artery. Top (B) and bottom (C) view of the harvested truncus. *Ao*. *V*, Aortic valve; *Pulm. V*, pulmonary valve.

**Figure 4:**
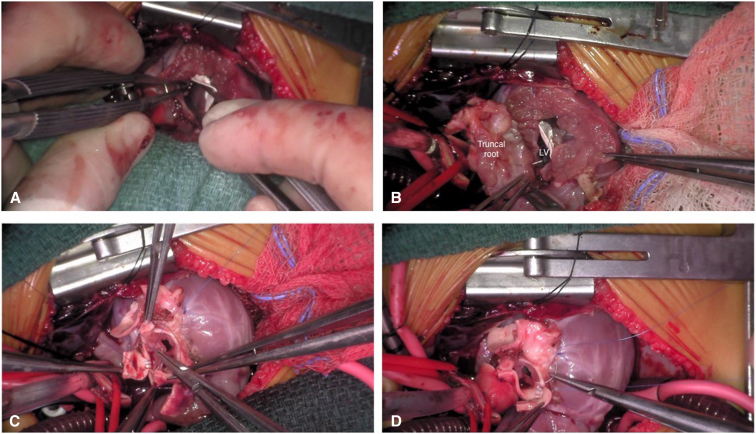
A, Ventricular septal defect patch and straight left ventricular outflow tract (*LVOT*). B, Suturing the half-turned truncal root to the LVOT. Suture lines during reimplantation should be full thickness when possible and always include the epicardium at a minimum. Using this technique has allowed us to avoid any significant postoperative bleeding. C and D, Coronary artery reimplantation. *LV*, Left ventricle.

**Figure E1:**
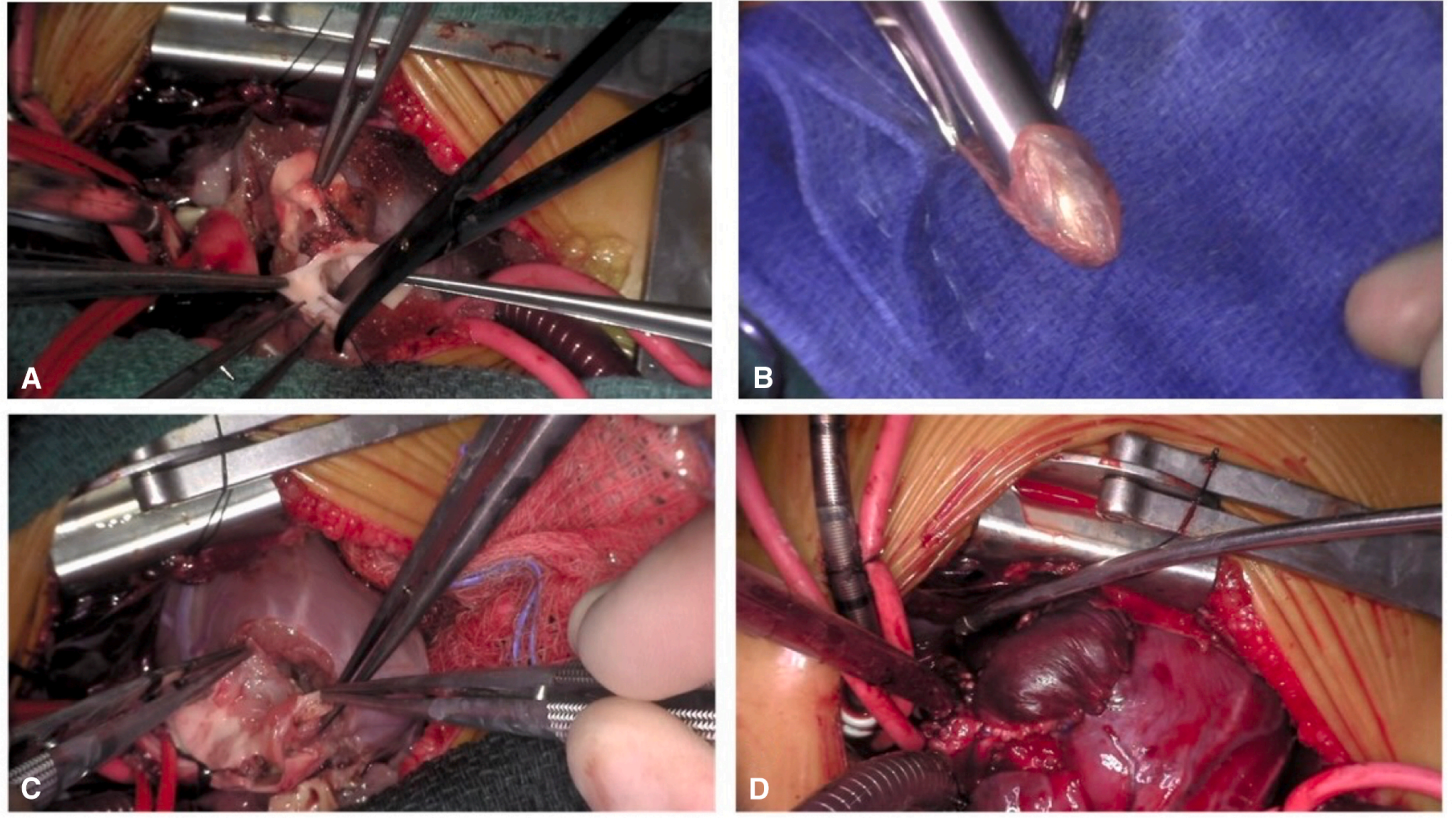
A, Opening of the pulmonary valve. B, Extracellular matrix right ventricular outflow tract (*RVOT*) patch with monocusp valve. New right ventricular outflow tract (C) with completed RVOT patch with the monocusp valve augmentation in place (D).

Operative techniques included standard cardiopulmonary bypass with bicaval cannulation, mild systemic hypothermia, and single-dose antegrade crystalloid cardioplegia with topical cooling, through a median sternotomy or resternotomy (for reinterventions). The operative procedure for HTTS has been described previously.^8^, ^9^, ^10^ We have made several modifications described here. Essentially, the ascending aorta is transected about 10 mm above the sinotubular junction and the pulmonary trunk is incised just proximal to the pulmonary bifurcation horizontally (Figure 1, *A* and *B*). Both coronary orifices are resected with a *U*-shaped incision (Figure 1, *C* and *D*). In the event of Type D coronary anatomy, the coronary artery should be mobilized along its proximal length to access the posterior aspect of the conus for the harvesting of the autograft. Great care must be taken to avoid coronary injury and allow for adequate muscle to remain for suturing of the autograft (Figure 2). The incision line is maintained a few millimeters from the aortic annulus, again to allow for adequate tissue for suturing of the autograft after the 180° rotation. Both ends of the infundibular septum are incised longitudinally toward the superior margin of the VSD. If the VSD is of the remote type, the infundibular septum is incised transversely because the incision is not extended to the VSD. The completely harvested truncal block includes the entire aortic and pulmonary valves (Figure 3, *B* and *C*). The VSD is closed using a teardrop or *U*-shaped patch. The width of the superior margin of the VSD patch is adjusted to the required length for the neoaortic annulus, which is calculated from the difference between the circumference of the aortic annulus and the LVOT (Figure 4, *A*). The truncal block is half-turned, or rotated, 180° horizontally, so that the aortic valve is positioned over the LVOT opening. The truncal block position should be carefully adjusted so that the left coronary artery cuff faces directly toward the coronary defect in the aortic wall. The posteriorly translocated aortic valve is anastomosed to the LVOT with a polypropylene running suture (Figure 4, *B*). Again, care must be taken, in Type D coronary anatomy, that the circumflex coronary artery is not injured during the suturing process (Figure 3, *A*). The coronary buttons are anastomosed to the corresponding aortic wall defects (Figure 4, *C* and *D*). The pulmonary bifurcation is translocated anteriorly (LeCompte maneuver) and the ascending aorta is shortened to prevent kinking due to posterior displacement. The ascending aorta is then reconstructed, and the distal main pulmonary trunk is anastomosed to the anteriorly translocated pulmonary bifurcation. If adequate pulmonary annular diameter and flexible pulmonary valve are obtained, only pulmonary commissurotomy is performed. The anterior wall of the PA is incised longitudinally through the anterior commissure in cases with a small pulmonary annulus or the midpoint of the anterior valve leaflet, and the anterior wall of the RVOT is covered with a monocusp fan-shaped CorMatrix patch with bulging sinus^10^ (Figure E1).

### Statistical Analysis

Data were analyzed using the SPSS version 22.0 software (IBM-SPSS Inc). Continuous variables were expressed as mean ± SD or as median and range, and categorical variables as numbers and percentages. The independent sample *t* test was used for comparative analysis between 2 groups for normally distributed data, the results of which were confirmed by the Kolmogorov-Smirnov test. A Fisher exact test was used for comparative analysis between independent groups for categorical variables. The long-rank test was used to estimate the statistical difference between the 2 groups of patients. Correlation analysis between variables was performed by Pearson rank correlation. Early mortality was defined as death during initial hospitalization or within 30 days of the procedure. Any deaths later than that were defined as late mortality.

## Results

Patients’ profiles are shown in Table 2. There were no early deaths; all patients survived the procedure. The groups did not differ significantly in gender, age/weight/height in complete repair, previous surgical or transcatheter procedures, postoperative length of stay, genetic/syndromic abnormalities, and crossclamp time during complete repair (Table 2). The mean cardiopulmonary bypass time was significantly longer for patients after HTTS (HTTS group, 264 ± 24 minutes vs Rastelli group, 174 ± 57 minutes; *P* = .01).

**Table 2:** Patient profiles (n = 11)

Patient	Diagnosis	Prior intervention	Age (mo)	Weight (kg)	Length of stay (d)	Outcome
HTTS group						
1	DORV/TGA/PS	None	31	14.5	7	Alive
2	DORV/TGA/PS	BTS	22	9.8	14	Alive
3	DORV/TGA/PS	None	4	8.3	8	Alive
4	DORV/TGA/PS	Central shunt	11	8.6	15	Late death
5	DORV/TGA/PS	None	14	8	7	Alive
Rastelli group						
6	TGA/PS	BTS	5.5	4	17	Reintervention
7	TGA/PS	BTS	12	8.1	18	Reintervention
8	DORV/TGA/PS	PDA stent	1.6	5.6	15	Alive
9	TGA/PS	None	13	7.1	7	Reintervention
10	DORV/TGA/PS	PDA stent	9	8	71	Reintervention
11	DORV/TGA/HRV/PS	None	16	10	49	Alive

*HTTS*, Half-turned truncal switch; *DORV*, double-outlet right ventricle; *TGA*, transposition of the great arteries; *BTS*, Blalock-Taussig shunt; *PS*, pulmonary stenosis; *PDA*, pattern ductus arteriosus; *HRV*, hypoplastic right ventricle.

The mean follow-up time was 5.2 ± 4.8 years (range, 1 month to 12 years). There was no significant difference between groups in follow-up time, PS gradient, degree of pulmonary or aortic insufficiency, or LVOT gradient (Table 3).

**Table 3:** Intraoperative and early postoperative variables comparison∗

Variable	Rastelli group (n = 6)	HTTS group (n = 5)	*P* value
CPB time (min)	174 ± 57	264 ± 24	.01
Aortic crossclamp time (min)	135 ± 58	182 ± 10	.11
Conduit diameter (mm)	13.7 ± 2.3	–	–
Length of stay (d)	29.5 ± 25	10.2 ± 4	.12
Follow-up time (y)	5.2 ± 4.8	4.6 ± 5.9	.85
Mortality	0	1 (20)	.40
Echocardiographic data at discharge			
Peak PS gradient (mm Hg)	10 ± 9.4	18.4 ± 19	.40
Degree of PI	1.7 ± 1.0	1.4 ± 1.1	.70
Degree of AI	0.5 ± 0.5	1 ± 0.7	.24
Peak LVOT gradient (mm Hg)	2.2 ± 0.8	3.7 ± 2.3	.25
Echocardiographic data at last follow-up			
Peak PS gradient (mm Hg)	31 ± 28	20 ± 16	.47
Degree of PI	2.1 ± 0.2	1.6 ± 1.1	.40
Degree of AI	1 ± 0.6	1 ± 0.7	1.00
Peak LVOT gradient (mm Hg)	2.3 ± 0.7	2.9 ± 1.8	.54

Values are presented as mean ± SD or n (%). *HTTS*, Half-turned truncal switch; *CPB*, cardiopulmonary bypass; *PS*, pulmonary stenosis; *PI*, pulmonary insufficiency; *AI*, aortic insufficiency; *LVOT*, left ventricular outflow tract.

∗ The independent sample *t* test was used for comparative analysis between 2 groups for normally distributed data, the results of which were confirmed by the Kolmogorov-Smirnov test. A Fisher exact test was used for comparative analysis between independent groups for categorical variables. The long-rank test was used to estimate the statistical difference between the 2 groups of patients. Correlation analysis between variables was performed by Pearson rank correlation.

There was 1 late death (HTTS group). Postoperatively, the patient was extubated but reintubated on postoperative day 2 for severe hypoxemia related to COVID-19 viral infection. She recovered from this and was discharged home in good health with normal biventricular function and no outflow tract obstruction. Several months later, the patient was readmitted due to bronchiolitis in the setting of rhino- and enterovirus infection. Echocardiography was performed that showed normal biventricular function. The following day the patient experienced cardiac arrest of unclear etiology and extracorporeal membrane oxygenation was initiated. The patient died due to multiorgan dysfunction a few days later. The overall survival rate was 90% at 10 years.

**Figure E2:**
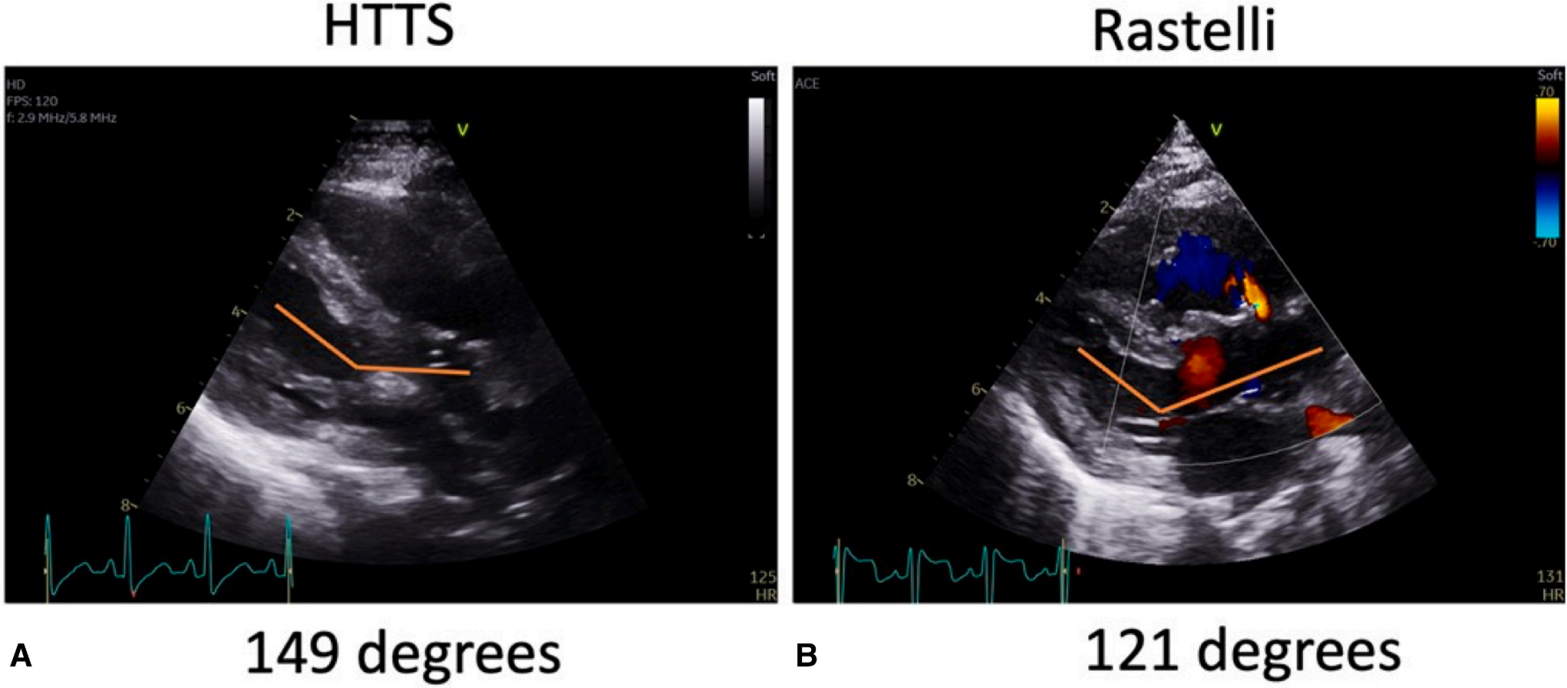
Echocardiographic imaging of the angle of the left ventricular axis to aorta in half-turned truncal switch (*HTTS*) (A) versus Rastelli (B) operations.

Nine patients were classified as New York Heart Association functional class I. Echocardiography at last follow-up revealed no ischemic segmental wall motion changes in any patients. Mean peak LVOT gradients were 2.3 ± 0.7 mm Hg in the Rastelli group and 2.9 ± 1.8 mm Hg in the HTTS group (*P* = .54). The LVOT in the HTTS patients had a much straighter pathway from the LV cavity to the ascending aorta as measured by 2-dimensional echocardiography (Figure E2). Aortic regurgitation was no more than mild in all patients. The freedom from reoperation rate for LVOT was 100%. Conduit reintervention was required in 4 patients from the Rastelli group and none in the HTTS group (Rastelli group, 67% vs HTTS group, 0%; *P* = .06).

Due to the small number of patients, power is lacking to provide a statistically significant difference. However, Rastelli operation appears to represent potential risk factor for reoperation on the RVOT.

## Discussion

The HTTS operation was originally described by Yamagishi and colleagues^9^^,^^10^ in 2003 for the treatment of TGA with VSD and important LVOT obstruction. The operation was designed to overcome the 2 major drawbacks of the Rastelli repair; that is, the eventual development of RVOT obstruction and the need for conduit change, and the development of LVOT obstruction due the long baffle required with a classic Rastelli repair.^1^^,^^5^^,^^6^ Over the years, different operations have been proposed to mitigate 1 or the other of these long-term complications.

The REV procedure as originally described by LeCompte and colleagues^2^ eliminates the need for a RV-PA conduit and the subsequent exchanges by advocating direct implantation of the main PA to the RV. However, the risk associated with LVOT obstruction due to a long intraventricular baffle and tunnel remains a limitation of the REV technique.^4^ The aortic translocation procedure, eponymously known as the Nikkaidoh operation, eliminates the need for the interventricular baffle and tunnel, by placing the aorta directly over the LV, thus straightening the LVOT. However, this operation still requires a RV-PA conduit, leaving the patient at risk for a lifetime of RVOT obstruction due to conduit stenoses, and the need for reinterventions (Table E1).

En bloc rotation of the outflow tracts, double root translocation, or the truncal half-turn operation all offer anatomic repair of transposition of the great arteries, VSD, and LVOT obstruction and closely related forms of double-outlet right ventricle. The main technical components include excision of the aortic and pulmonary roots and rotation of the truncal root 180° followed by reimplantation. The VSD patch closes the VSD and straightens and enlarges the LVOT. Mair and colleagues^13^ recently reported on their experience with 27 patients who underwent truncal half-turn from 2003 to 2019 and the authors found that two-thirds of the pulmonary valves were able to be preserved. Their group also demonstrated growth of the aortic and pulmonary roots in these patients. They concluded that although it is a technically demanding operation, morbidity and mortality did not differ from other forms of palliation and the need for reoperations and reinterventions was very low.^13^

In 2021, Hongu and colleagues^8^ compared the HTTS operation or half turned truncal switch operation with conventional treatments for TGA with VSD and LVOT obstruction and used 4-dimensional flow magnetic resonance imaging to describe anatomic and physiologic attributes of the LVOT. They believed the advantages of the HTTS operation were a wide straight LVOT, low energy loss, and low wall shear stress in the ascending aorta. In addition, they maintain that the possibility of pulmonary outflow tract growth potential is preserved.^8^ In reviewing the care and treatment of a cohort of patients treated at our institution we noted several conclusions. None of the patients required reoperation or reintervention for LVOT obstruction. The LVOT in the HTTS patients had a much straighter pathway from the LV cavity to the ascending aorta as measured by 2-dimensional echocardiography (Figure E2). Given the importance of this straight pathway to decrease wall stress and turbulence and potentially decrease the incidence of LVOT obstruction and preserve ventricular function over the long term, this is a significant advantage of the HTTS.

Limitations of the truncal half-turn technique have been described and include important coronary artery patterns that would prohibit the performance of the operation. These include any type of coronary arrangement that would put the coronary arteries in the path of the dissection and excision of the truncal root. Because the coronary arteries typically arise from the posterior facing sinuses, the HTTS operation is usually indicated for dextro-TGA with the most common coronary artery pattern which is Yacoub type A. In the instance of Yacoub type D, where the left circumflex coronary artery courses posterior to the pulmonary artery, the HTTS technique is not an absolute contraindication; however, caution must be taken to leave enough distance between the circumflex coronary artery and pulmonary annulus to accommodate both resection and reanastomosis to the half-turned truncal block.^14^ In contrast, several authors have described important anterior branches crossing the RVOT to be a contraindication.^10^^,^^15^ In our series, we also did not perceive the Type D arrangement of the coronary arteries to be a contraindication (Figure 3, *A*). With careful dissection and mobilization of the proximal coronary arteries, the truncal root can be safely excised, the half-turn completed, and the root sewn back in place avoiding important coronary artery injury or insufficiency.

Although the use of HTTS operation has recently been described by Asada and colleagues^16^ in situations with side-by-side great vessel arrangement, we have not embarked on this path of use and have used the Rastelli operation in those patients. Our experience thus far with the HTTS technique has made the use of it in side-by-side great vessel arrangements appealing and it likely warrants further consideration in this group at our institution.

Overall, the HTTS operation provides an excellent palliation for patients with TGA and double-outlet right ventricle with VSD accompanied by LVOT obstruction. It compares very favorably with other forms of palliation by providing a straight and unobstructed LVOT and an unobstructed RVOT without the need for an RV-PA conduit. Additionally, the HTTS technique provides the potential for long-term growth of the outflow tracts and limits the possibility of obstruction. We believe the highly technical nature of the procedure is far outweighed by these benefits and we will continue to use and expand its application to various types of outflow tract and coronary anatomical variations. At our institution, HTTS is considered the treatment of choice for these patients when not contraindicated by patient anatomy. These patients will continue to be followed longitudinally, and we hope eventually to report on this progress. Time will tell if this can be more broadly recommended and applied to our specialty as a whole and adapted by other centers.

## Conflict of Interest Statement

The authors reported no conflicts of interest.

The *Journal* policy requires editors and reviewers to disclose conflicts of interest and to decline handling or reviewing manuscripts for which they may have a conflict of interest. The editors and reviewers of this article have no conflicts of interest.

## Figures and Tables

**Table E1:** Long-term risks by procedure type

Operation	RV-PA conduit	Interventricular tunnel	Risk for RVOT obstruction	Risk for LVOT obstruction
Rastelli	Yes	Yes	+++	++
REV	No	Yes	−/+	++
Aortic translocation	Yes	No	+++	–
Truncal half-turn	No	No	–	–

+++, Significant; ++, moderate; –/+, equivocal; –, none.

*RV-PA*, Right ventricle to pulmonary artery; *RVOT*, right ventricular outflow tract; *LVOT*, left ventricular outflow tract; *REV*, réparation a l'étage ventriculaire.
